# Ligand-dependent dynamics of retinoic acid receptor binding during early neurogenesis

**DOI:** 10.1186/gb-2011-12-1-r2

**Published:** 2011-01-13

**Authors:** Shaun Mahony, Esteban O Mazzoni, Scott McCuine, Richard A Young, Hynek Wichterle, David K Gifford

**Affiliations:** 1 Computer Science and Artificial Intelligence Laboratory, Massachusetts Institute of Technology, 32 Vassar Street, Cambridge, MA 02139, USA; 2 Departments of Pathology, Neurology, and Neuroscience, Center for Motor Neuron Biology and Disease, Columbia University Medical Center, 630 West 168th St, New York, NY 10032, USA; 3 Whitehead Institute for Biomedical Research, Nine Cambridge Center, Cambridge, MA 02142, USA

## Abstract

**Background:**

Among its many roles in development, retinoic acid determines the anterior-posterior identity of differentiating motor neurons by activating retinoic acid receptor (RAR)-mediated transcription. RAR is thought to bind the genome constitutively, and only induce transcription in the presence of the retinoid ligand. However, little is known about where RAR binds to the genome or how it selects target sites.

**Results:**

We tested the constitutive RAR binding model using the retinoic acid-driven differentiation of mouse embryonic stem cells into differentiated motor neurons. We find that retinoic acid treatment results in widespread changes in RAR genomic binding, including novel binding to genes directly responsible for anterior-posterior specification, as well as the subsequent recruitment of the basal polymerase machinery. Finally, we discovered that the binding of transcription factors at the embryonic stem cell stage can accurately predict where in the genome RAR binds after initial differentiation.

**Conclusions:**

We have characterized a ligand-dependent shift in RAR genomic occupancy at the initiation of neurogenesis. Our data also suggest that enhancers active in pluripotent embryonic stem cells may be preselecting regions that will be activated by RAR during neuronal differentiation.

## Background

Cellular competence, fate determination, and differentiation are influenced by the external signals cells receive. While these external signals can take the form of steroid hormones, protein growth factors, or other molecules, their presence is typically communicated by signal-responsive transcription factors (TFs). The effect of a signal on gene expression, and ultimately on cell fate, depends on where such TFs bind to the genome. Therefore, understanding how signal-responsive TFs are integrated into a dynamic cellular context will further our knowledge of the mechanisms guiding the acquisition of specific cellular identities.

In the developing neural tube, retinoid signaling initiates neural differentiation [1], specifies caudal hindbrain and rostral cervical spinal identity [2,3], and controls patterning and differentiation of spinal motor neurons and interneurons [4-6]. Retinoic acid (RA) is the most commonly used neuralizing agent during *in vitro *embryonic stem (ES) cell differentiation since exposure to it results in a rapid transition from pluripotent embryoid bodies to committed neuronal precursors. The response to RA during neuronal development is mediated by the action of retinoic acid receptor isoforms (collectively abbreviated here as RARs). It has been proposed that RARs are constitutively bound to target sites in the absence of retinoids [7], recruiting co-repressors such as Ncor1 and Ncor2 [8]. In the presence of the retinoid ligand, RAR (often heterodimerized with RXR) recruits co-activators (Ncoa1 and Ncoa2), p300, and core components of the transcriptional machinery [7]. However, the proposed independence of RAR binding from the presence of the ligand has only been confirmed at a small number of sites.

While some characterization of RAR genomic binding has recently been carried out in mouse ES and human breast cancer cell lines [9-11], it is unknown which genes are targeted by RAR during neurogenesis, and how RAR binding targets are selected. Chromatin accessibility and protein cooperativity may both play roles in restricting the cohort of bound locations under a given set of cellular conditions. For example, in human breast cancer cell lines, RAR binding is highly coincident with the binding of estrogen receptor (ER)α, FoxA1, and Gata3 [10,11], and FoxA1 is required for RAR recruitment [10]. Recent work has demonstrated that TF binding also correlates with nucleosome-free regions [12], certain histone modifications [13-17], and the occupancy of other regulatory proteins [18,19] in the same cellular conditions. It is not known how these relationships extend through developmental time at individual enhancers. Enhancers may be entirely developmental stage-specific, in which case the sites bound by a regulator in one developmental stage should not be coincident with the sites bound by a subsequent stage-specific TF. Alternatively, enhancers may be reused across developmental time, and the occupancy patterns of regulatory proteins or epigenetic markers may anticipate the future binding of newly activated TFs during differentiation [20,21]. Determining the dynamics of RAR binding during early neuronal development may therefore yield insight into the precise temporal response of cells to retinoid signaling and how enhancers are organized to facilitate this response.

In this study, we examine the genome-wide binding of RARs during RA induced differentiation of ES cells into spinal motor neurons [22]. Retinoid signaling initiates the transition from pluripotency to neurogenesis in this model system, and provides rostro-caudal information to developing motor neurons. By profiling the binding of active RAR isoforms in both the presence and absence of retinoid signaling, we observe that only a small subset of sites are constitutively bound. An additional set of sites is bound only in the presence of RA, and the existence of this set provides a convenient opportunity to examine how pre-RA occupied and post-RA occupied sites correlate with the relatively well-characterized regulatory network in mouse ES cells. We find that binding information for ES cell TFs and other regulatory proteins accurately predicts both constitutive and exclusively post-RA RAR binding. The binding of core ES cell regulators is highly correlated with pre-RA bound RAR sites, slightly less correlated with post-RA bound RAR sites, and much less correlated with the binding of other TFs in further differentiated tissues, arguing that the active regulatory network may be one of the most important determinants of TF binding.

## Results

### RAR ChIP-seq profiles direct genomic interactions during early differentiation

**Figure 1:**
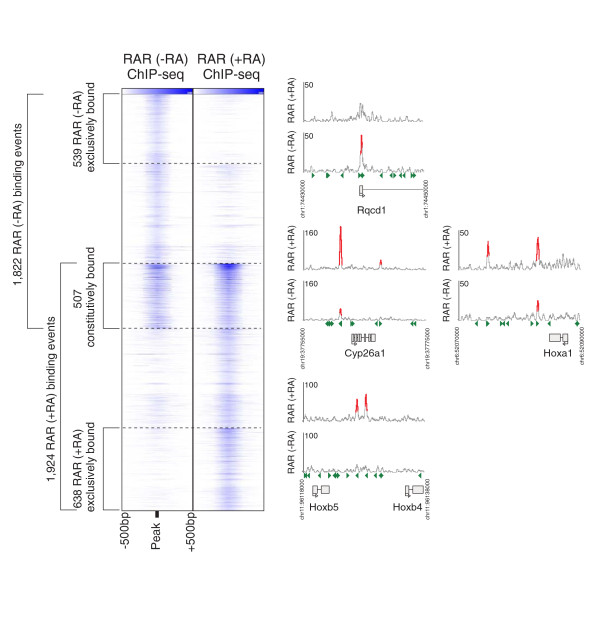
**RAR binding shifts in response to RA exposure**. **(a) **The plots in the two leftmost columns show enrichment over all 1,822 pre-RA and 1,924 post-RA RAR binding sites (± 1 kbp over the binding site), where the blue shading corresponds to the ChIP-seq read count in the region. **(b) **Examples of constitutive and ligand-specific RAR binding at four loci (Rqcd1, Cyp26a1, Hoxa1, Hoxb4/Hoxb5).

Using a pan-RAR antibody, we profiled the genome-wide occupancy of RAR isoforms in differentiating embryoid bodies after 8 hours of exposure to RA, finding significant ChIP-seq enrichment at 1,924 sites. We also profiled RAR occupancy in the same developmental stage but in the absence of retinoid signaling, finding 1,822 sites of significant enrichment. A number of previously characterized retinoic acid response elements (RAREs) were observed to be bound in both conditions, including RAREs at *Rarb*, *Hoxa1*, and *Cyp26a1 *(Figure 1) [23]. A recent promoter-focused ChIP-chip study of RAR in mouse embryonic stem cells [9] suggested that few RAR binding sites contained 'direct-repeat' hormone response elements. In contrast, we find that high-similarity hormone response element motifs occur at RAR ChIP-enriched sites at a higher rate than that observed in published ChIP-seq studies of other nuclear hormone receptors such as ERα, Esrrb, and Nr5a2 [10,24-26] (Additional file 1). The most frequent motifs at our enriched sites are the direct-repeat motifs with spacers of 5 bp or 2 bp (DR5 and DR2, respectively; Additional file 1), which RAR is known to preferentially bind [23,27]. The binding events with the highest ChIP-enrichment are more likely to contain high-similarity matches to the DR5 and DR2 motifs (Additional file 2), suggesting that many of the most enriched sites represent direct RAR-DNA binding events.

### RAR binding shifts in response to RA exposure

In contradiction to the model of RAR constitutively binding to its targets [7], only 507 of the predicted RAR binding events are significantly enriched both in the presence and absence of retinoid exposure, where significant enrichment is defined by our binding event detection methodology (see Materials and methods). Figure 1 presents a clustergram of all sites bound before or after RA exposure, and is arranged according to the pattern of enrichment across both conditions. As the figure indicates, we need to be cautious when determining if a site is bound exclusively in one condition. For instance, some sites display similar enrichment levels across both conditions, but this enrichment level is only deemed significant in one condition (that is, it falls below the significance threshold in the other condition). After further analysis, we define a set of 638 sites that are bound exclusively in the presence of retinoid signaling, as they are not significantly enriched in the absence of RA exposure (compared with control), and their levels of ChIP-seq enrichment are significantly different in the presence and absence of RA (see Materials and methods). Conversely, at least 539 sites are bound only in the absence of retinoid exposure.

Intriguingly, some of the shift in RAR binding sites may be explained by a ligand-dependent shift in RAR's binding preference. Sites bound only in the absence of RA contain more direct repeat motifs with 0-bp or 1-bp spacers than sites bound only in the presence of RA (Additional files 3 and 4). Prior studies have shown that such motif configurations can be bound by RAR [28,29]. On the other hand, sites bound exclusively in the presence of RA contain more DR5 motifs. These direct repeat motifs are amongst the set of sequence features that have the most significant difference in occurrence frequency between RAR sites bound exclusively in the presence or absence of retinoid signaling (Additional file 5). However, only approximately 14% of exclusively pre-RA sites contain high similarity matches to the DR0 or DR1 motifs, while only 13% of exclusively post-RA sites contain high similarity DR5 motifs. Therefore, a potential shift in RAR's direct binding preference offers only a partial explanation for the observed condition-exclusive binding patterns.

By comparing the relative occurrence of all known TF binding motifs in each condition-exclusive set, we also find that exclusively post-RA sites contain significantly more E-box and ETS-family motifs than exclusively pre-RA sites (Additional file 5). Exclusively post-RA sites also contain more instances of a palindromic motif with consensus sequence 'TCTCGCGAGA'. It is not known which proteins may interact with this motif, although the motif is over-represented in mammalian promoter regions [30], and has recently been characterized as a regulatory sequence [31]. The observation of these over-represented secondary motifs suggests that some of the exclusively post-RA binding sites may occur due to ligand-dependent interactions between RAR and cofactors, or some may potentially represent indirect binding events caused by enhancer-promoter looping. Most of the motifs with significantly higher relative frequency in the exclusively pre-RA sites are related to DR0 or DR1 patterns.

### A compact retinoid response is directly mediated by RAR

**Figure 2:**
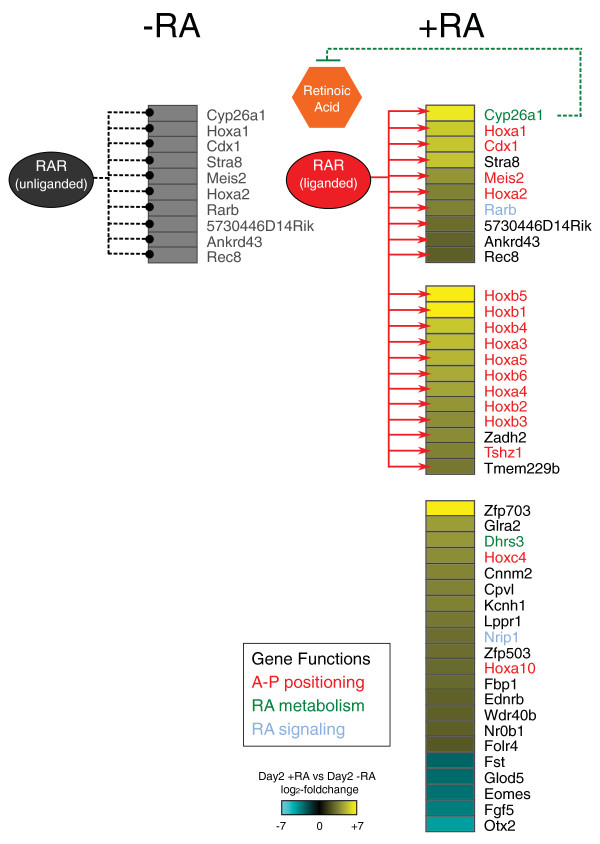
**Direct binding of RAR mediates the initial response to RA during early neurogenesis**. Genes with more than five-fold differential expression after 8 hours of RA exposure are listed. RAR binds to many of the up-regulated genes, with binding more likely for greater degrees of up-regulation. Red arrows indicate post-RA RAR binding within 20 kbp of the gene. Black dashed lines indicate pre-RA RAR binding within 20 kbp. Three functional groups of genes are indicated by coloring the gene names. Information for all more than two-fold differentially expressed genes is tabulated in Additional file 2.

In order to determine which RAR binding sites are associated with transcriptional regulation, we characterized the early transcriptional response to retinoid signaling. Despite the dramatic consequences initiated by RA exposure, microarray-based gene expression analysis reveals that only 96 genes are differentially expressed given 8 hours of RA exposure (more than two-fold change, *P *< 0.01; Additional file 6). Of these, 81 genes are up-regulated. The most prevalent theme in the expression response is the acquisition of rostro-caudal identity; 12 anterior Hox genes are significantly up-regulated, along with the Hox co-factors *Meis1*, *Meis2*, *Pbx2*, and other positioning genes such as *Tshz1 *and *Cdx1*. While RARβ is up-regulated, the response also attenuates retinoid signaling via the induction of retinoid metabolism genes (*Cyp26a1*, *Dhrs3*, *Rbp1*) and a repressor of RAR, *Nrip1 *[32]. Thirty-five significantly up-regulated genes are within 20 kbp of a post-RA RAR binding event, including many of the most differentially expressed genes (Figure 2; Additional file 6). Exclusively post-RA RAR targets are no less associated with differential expression than the constitutively bound targets; while 20 significantly up-regulated genes are nearby constitutively bound RAR sites, 15 up-regulated genes are only bound after RA.

### RAR binding is associated with RNA polymerase II initiation

**Figure 3:**
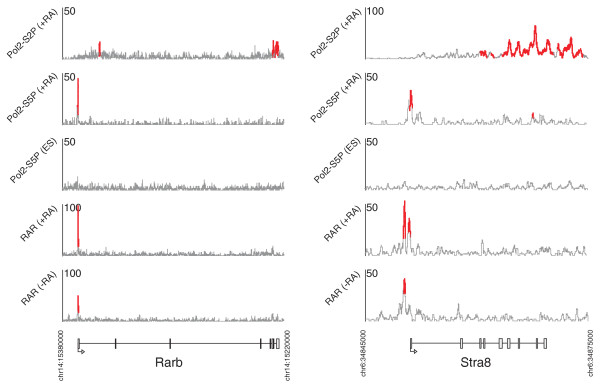
**Constitutive RAR binding without ES cell-poised Pol2 at Stra8 and Rarb**. RAR is constitutively bound at these targets, but no enrichment of poised/initiating polymerase (Pol2-S5P) is observed in ES cells at these loci. Within 8 hours of retinoid exposure, the initiating and elongating forms of Pol2 are recruited to these genes.

The set of RAR binding sites near differentially expressed genes represents a small proportion of the total complement of post-RA RAR binding sites. It is likely that many other RAR binding sites play regulatory roles during the retinoid response that are not apparent from microarray-based differential expression analysis. We used ChIP-seq to characterize RNA polymerase II (Pol2) initiation (as signified by Pol2 CTD serine 5 phosphorylation, Pol2-S5P [33-35]) and elongation (as signified by Pol2 CTD serine 2 phosphorylation, Pol2-S2P [33-35]) after 8 hours of RA exposure. We identified 3,409 significant Pol2 initiation events, of which 424 were within 5 kbp of post-RA RAR binding events. Of these RAR-associated Pol2-S5P events, 402 (95%) are within 1 kbp of the transcription start sites, or within the gene body, of 269 known genes and non-coding RNAs. Significant enrichment of Pol2-S2P is observed within or at the 3' end of 214 genes (80%) bound by RAR and Pol2-S5P, demonstrating that many of these genes are actively transcribed post-RA (for example, see Figure 3). Therefore, the correlation between RAR binding and Pol2 initiation and elongation suggests that RAR may play a wider role in driving and maintaining transcription beyond that observed from microarray-based differential expression analysis. We again find no evidence that exclusively post-RA RAR binding sites are less associated with Pol2 initiation than constitutively bound sites; both sets of sites are coincident with Pol2-S5P events at similar rates.

A proposed model of RAR functionality suggests that it acts as a transcriptional repressor in the absence of RA signaling, and becomes an activator after ligand binding [7]. To assess the dynamics of RAR's interactions with Pol2, we compare the post-RA Pol2 ChIP-seq profiles with Pol2-S2P and Pol2-S5P ChIP-seq data from the pluripotent state [36]. Of the 424 RAR-associated Pol2-S5P events characterized post-RA, the majority (390) are also enriched for Pol2-S5P in the pluripotent state. The pre-RA pattern of RAR binding does not seem to affect the behavior of Pol2 at these sites; both constitutive and exclusively post-RA RAR binding sites are coincident with constitutive Pol2 initiation events at similar rates. From the 214 RAR-bound genes that displayed enrichment for both initiating and elongating Pol2 after RA exposure, 54 (25%) also display evidence of Pol2 elongation in the pluripotent state. Genome-wide, we find a set of only 27 significant Pol2-S5P initiation events that are bound by Pol2 after RA exposure but show no evidence of enrichment in pluripotent cells. Only 11 of these events are near RAR binding events. Surprisingly, this compact set of RAR targets for which Pol2 is not poised in pluripotent cells includes *Hoxa1*, *Cyp26a1*, *RARb*, and *Stra8 *(for example, see Figure 3). Therefore, these critical RA-responsive genes are constitutively bound by RAR, but Pol2 is only recruited to their promoters after RA exposure.

In summary, our examination of potential interactions between RAR and Pol2 before and after retinoid exposure adds complexity to the proposed model of RAR functionality. Only a small set of important retinoid targets fit the simple model of RAR recruiting Pol2 to the transcription start site only after RA exposure. Many more RAR target genes already have poised Pol2 before retinoid signaling, regardless of whether RAR is constitutively bound. A further set of bound genes is already being actively transcribed before RA exposure.

### RAR binding is associated with ES cell regulatory state

DNA-binding preference alone is not sufficient to explain the specificity of RAR's post-RA genomic occupancy. At least 150,000 high-similarity matches to the DR2 and DR5 motifs do not display significant RAR binding either before or after RA exposure. One possibility is that RAR bound sites are distinguished by their chromatin structure profiles and the occupancy of other regulatory proteins in the surrounding genomic region. To assess the regulatory state of RAR binding sites, we compare constitutively bound sites (by definition occupied both post-RA and in the preceding pluripotent state) to published ChIP-seq data in mouse ES cells, including data for multiple TFs, co-factors, histone modifications, and chromatin modifying proteins [24,37-41].

**Figure 4:**
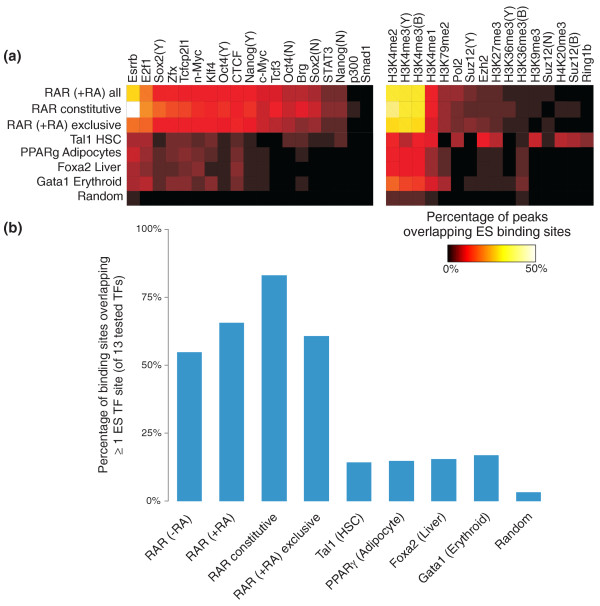
**RAR binding sites are coincident with ES cell transcription factor binding and H3K4 methylation**. **(a) **Percentages of binding sites within 200 bp of ES cell binding events. Coincidence rates between 10,000 random genomic locations and ES cell binding events are shown for reference. In cases where the same protein was profiled by multiple labs, we denote the source using the following abbreviations: B, Bernstein lab [38-40]; N, Ng lab [24]; Y, Young lab [37]. **(b) **Rates of post-ES cell binding sites where at least one ES cell TF binding site (of 13 profiled TFs) is within 200 bp. HSC, hematopoietic stem cell.

**Figure 5:**
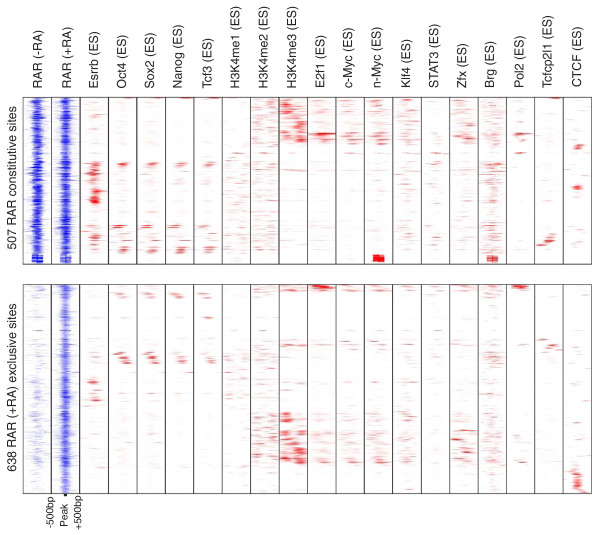
**Both constitutively bound and exclusively post-RA RAR binding sites are coincident with ES cell regulatory events**. Line-plot clustergram of ChIP-seq enrichment in 1-kbp windows centered on 1,924 post-RA RAR binding sites. Color shading denotes scaled ChIP-seq read depth (see Materials and methods).

We observe that the locations of constitutively bound RAR binding sites are highly coincident with the binding sites of many regulatory proteins in ES cells (Figures 4a and 5). While only 3% of randomly selected sites are within 200 bp of at least one ES cell TF binding site, 83% of constitutively bound RAR sites display the same proximity (Figure 4b). Surprisingly, the associations are not limited to general TFs; many exclusively post-RA RAR sites are coincident with the binding sites of core ES cell state regulators, such as Esrrb and Oct4.

RAR must recognize the sites bound exclusively post-RA after the established ES cell pluripotent regulatory state has begun to respond to RA exposure. According to the hypothesis that all developmental enhancers are epigenetically marked at the earliest stages of development [20,21], RAR will bind post-RA to sites that are already bound by other regulators in ES cells. Alternatively, RAR may recognize unbound developmental enhancers that are specific to neuronal fate. We find that 61% of exclusively post-RA RAR binding sites are within 200 bp of at least one known ES cell TF binding site (Figure 4b). Thus, the observed associations between RAR and ES cell TF binding sites suggest that RAR binds to some sites that were bound by stage-specific TFs in the earlier pluripotent state, even at sites to which RAR itself was not bound in that stage. However, the associations between ES cell binding sites and exclusively post-RA RAR sites are less than those with constitutively bound RAR sites, and thus our observations are not fully consistent with the hypothesis that all developmental enhancers are marked in ES cells.

To further examine the relationships between ES cell regulatory state and later developmental enhancers, we analyzed data from published ChIP-seq experiments performed in unrelated adult or late differentiation cell types: Foxa2 in liver [17], Gata1 in erythroid cells [42], Tal1 in hematopoietic stem cells [43], and peroxisome proliferator activated receptor (PPAR)γ (another nuclear hormone receptor) in adipocyte differentiation [25]. While all of these stage-specific TFs bind to the same regions as ES cell TFs at a higher rate than expected by chance (Figure 4a), none of them approaches the rate of overlap observed for RAR during early differentiation. Therefore, the relationships between RAR and ES cell TFs do not merely result from all possible enhancers being unveiled by ES cell ChIP-seq data.

### ES cell TF binding predicts post-RA RAR binding

**Figure 6:**
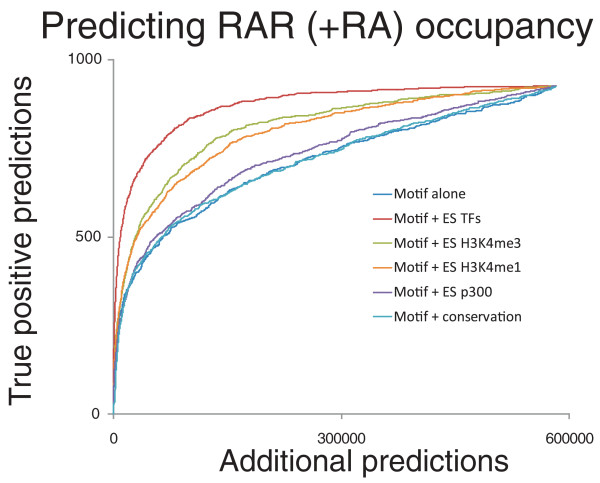
**ChIP-seq data improves motif specificity**. The true positive and additional prediction rates are shown when predicting post-RA RAR binding sites by ranking sites according to motif similarity or when combining motif information with various other data sources (see Materials and methods).

The observed relationships between RAR binding and earlier binding events suggest that TF binding information from ES cells can be used to predict where signaling TFs will bind in a proximal developmental state. Predicting if a motif sequence will be bound based on motif similarity alone leads to high rates of additional predictions (Figure 6) [44]; for a motif similarity threshold with which we can correctly predict 500 post-RA bound RAREs, we also predict that approximately 65,000 additional sites should be bound. Recent reports demonstrate the use of co-temporal histone modification ChIP-seq data for predicting TF binding to motif sequences [14,16,45]. We can similarly combine the motif-similarity score with a score based on the sum of normalized read counts from ES cell TF ChIP-seq experiments in 500-bp windows around the sites (see Materials and methods). As shown in Figure 6, this combined score significantly decreases the rate of additional predictions for a given true-positive rate. Using the combined motif and ES cell TF score, we reduce the number of additional predictions 85% (to approximately 9,600) while correctly predicting 500 bound RAREs. We find that ES cell TF binding data outperforms conservation, ES cell p300 ChIP-seq data, and ES cell H3K4 methylation data in predicting which RARE motifs will be bound (Figure 6).

Note that the improvement in predictive performance described above is achieved with a naïve approach that assumes all ES cell TF data sources are equally informative for post-RA RAR binding. We can compare the predictive performance of ES cell TF data sources to that of histone modification information by training a supervised classification technique to classify sites as bound or unbound. Specifically, we trained support vector machines (SVMs) to discriminate between sites that are bound by RAR and a negative set of 10,000 unbound sites. As shown in Table 1, test set SVM performance is highest when making use of all available ES cell data. SVMs trained using the same ES cell data sources perform worse when predicting PPARγ binding in adipocytes or Foxa2 binding in liver (Table 1).

**Table 1:** Motif occupancy classification performance using ES cell ChIP training data

Binding sites	All ES cell experiments	ES cell TF experiments	ES cell histone modifications
RAR (constitutively bound)	0.96	0.92	0.81
RAR (post-RA exclusively bound)	0.81	0.77	0.73
PPARγ (adipocytes)	0.62	0.58	0.53
Foxa2 (liver)	0.63	0.56	0.50

Performance is measured as receiver operating characteristic (ROC) area under curves for SVMs trained to discriminate between significant binding sites and randomly selected unbound locations.

Interestingly, our SVM results suggest that the ES cell TF binding landscape is more informative than ES cell histone modification data when predicting the genomic locations that are bound by signal-responsive TFs. SVMs that are trained using only ES cell TF binding data offer higher classification performance of bound sites than SVMs that are trained using only ES cell histone modification data. This observation holds true when predicting sites that are only bound by RAR before or after RA exposure.

## Discussion

By profiling the dynamics of RAR occupancy at the initiation of neurogenesis, we have characterized a ligand-dependent shift in binding targets. This shift in binding targets is relevant to RAR's role in gene regulation, as both constitutively and exclusively post-RA bound sites are associated to a similar degree with gene expression and polymerase recruitment. Recent analyses of RAR binding profiled genome-scale occupancy only in the presence of retinoids, and thus did not observe a ligand-dependent shift in binding [9-11]. Indeed, on the basis of a small number of ChIP-quantitative PCR experiments, Delacroix *et al. *[9] suggested that most RAR binding sites are occupied both in the presence and absence of retinoids.

Some of RAR's shift in binding may be explained by ligand-dependent binding preference or ligand-dependent interactions between RAR and co-activators or co-repressors. In addition, a mixture of RAR isoforms is active at the initiation of neurogenesis, and changes in the composition of this mixture may lead to changes in binding occupancy. For example, RARβ is activated after retinoid exposure, and may have different binding preferences or cofactor interactions from the isoforms active in the absence of RA (RARγ and RARα). Preliminary evidence suggests that the pan-RAR antibody has limited affinity for RARβ, as we have not had success using this antibody for ChIP experiments at later points in development when RARβ becomes the dominant isoform (data not shown). However, given the panRAR antibody vendor specifications, we cannot exclude the possibility that some of the exclusively post-RA binding sites may be attributed to RARβ binding.

We have also found that the binding sites of RAR after RA signaling are extensively associated with the binding of other regulatory proteins in the temporally preceding pluripotent environment. Furthermore, we have demonstrated that we can accurately predict where RAR will bind in the genome given knowledge of the preceding regulatory state. The apparent dependence of RAR binding on prior cellular state suggests that the response of differentiating cells to external signals may be context and developmental-stage dependent, with some future binding events being potentiated by current genomic occupancy patterns.

The causal relationships underlying the association between RAR binding and the ES cell regulatory network remain unclear, so we can only summarize possible explanations for the observed data. ChIP-seq data from ES cells may provide a read-out of accessible regions of the genome, thereby indicating which regions are amenable to TF binding in that environment. Since the predictive capacity of ES cell regulatory data decreases with temporal distance from ES cell state (Table 1), we do not believe that ES cell ChIP-seq data merely serves as an indicator of all enhancers that may be bound under any condition or cell type. Rather, the regions bound by regulatory proteins in a given developmental stage may be more likely to remain accessible for TF binding in a related future stage. Direct cooperation between RAR and TFs active in ES cells may also account for some coincident binding sites. Of all tested data sources, Esrrb binding in ES cells is the most correlated with RAR occupancy before and after RA exposure. Esrrb is an orphan nuclear receptor that binds to hormone response element motifs. It is therefore possible that Esrrb heterodimerizes or otherwise directly cooperates with RAR at direct repeat hormone response element (HRE) motifs, facilitating stable binding events before and/or after RA signaling. However, direct interactions between Esrrb and RAR are not required for cooperativity to arise. For example, Esrrb could maintain chromatin accessibility at some direct repeat HREs until RAR binds after retinoid exposure. All of RAR's associations with ES cell core regulators cannot be explained by Esrrb occupancy alone; as shown in Figure 5, many RAR binding sites are associated with the binding of ES cell TFs other than Esrrb.

The observation that RAR binding is correlated with the occupancy of other regulatory proteins is supported by other recent ChIP studies of RAR. Delacroix *et al. *[9] demonstrate cell-type specific RAR occupancy in mouse ES cells and mouse embryonic fibroblasts, which correlates with cell-type-specific H3K4me3 patterns. Both Hua *et al. *[10] and Ross-Innes *et al. *[11] show that RAR and ERα colocalize at many regions in a human breast cancer cell line (MCF-7). Hua *et al. *[10] also demonstrate that many RAR and FoxA1 binding sites coincide in MCF-7 cells, and that RAR binding is decreased at such sites when FoxA1 is knocked down. Therefore, RAR may preferentially bind to RARE motifs that are made accessible by the binding of other TFs or chromatin modifying proteins.

A number of previous studies have demonstrated that certain regulatory information may be used to predict co-temporal TF occupancy. For example, enrichment of p300 [18], H3K4me1 [17,45], H3K4me3 [15,45], and regions of open chromatin (as assayed by DNaseI hypersensitivity [12,46]) have each been correlated with the binding of TFs in ES cells and other tissues. Ours is the first demonstration that regulatory information in a given cell type may be used to predict future TF binding events. Furthermore, the markers examined in the previous studies are typically associated with active enhancers. In our study, we use all available information to predict any RAR binding event, regardless of its association with transcription. Our rationale is that binding events that do not produce co-temporal transcription are not necessarily neutral, especially in the context of differentiation. For example, binding events that do not produce transcription under one set of conditions may disrupt chromatin structure enough to allow different proteins to bind to proximal sites during a future developmental stage.

## Conclusions

We have described a compact transcriptional response to RA at the initiation of neurogenesis, which may be potentiated by associations between RAR and earlier regulatory events. As more regulatory data are collected from a greater diversity of cell types and developmental stages, it will be of interest to further elucidate temporal dependencies between the genomic occupancy of regulatory proteins. Indeed, exploring such temporal networks of binding events may lead to greater understanding of the influences on cell fate during differentiation.

## Materials and methods

### Cell culture and motor neuron differentiation

ES cells were differentiated as previously described [22]. Briefly, ES cells were trypsinized and seeded at 5 × 10^5 ^cells/ml in ANDFK medium (Advanced DMEM/F12:Neurobasal (1:1) medium, 10% knockout-SR, Pen/Strep, 2 mM L-glutamine, and 0.1 mM 2-mercaptoethanol) to initiate formation of embryoid bodies (day 0). Medium was exchanged on days 1, 2 and 5 of differentiation. Patterning of embryoid bodies was induced by supplementing media on day 2 with 1 μM all-*trans*-RA (Sigma, St. Louis, MO, USA) and 0.5 μM agonist of hedgehog signaling (SAG, Calbiochem, La Jolla, CA, USA). For ChIP experiments, the same conditions were used but scaled to seed 1 × 10^7 ^cells on day 0.

### Expression analysis

Total RNA was extracted from ES cells or embryoid bodies using Qiagen RNAeasy kit (Qiagen, Valencia, CA, USA). For quantitative PCR analysis, cDNA was synthesized using SuperScript III (Invitrogen, Carlsbad, CA, USA) and amplified using SYBR green brilliant PCR amplification kit (Stratagene, La Jolla, CA, USA) and Mx3000 thermocycler (Stratagene). For GeneChip expression analysis, RNA was amplified using Ovation amplification and labeling kit (NuGen, San Carlos, CA, USA) and hybridized to Affymetrix Mouse Genome 430 2.0 microarrays. Expression microarray experiments were performed in biological triplicate for each analyzed time point. Arrays were scanned using the GeneChip Scanner 3000. Data analysis was carried out using the affylmGUI BioConductor package [47]. GC Robust Multi-array Average (GCRMA) normalization [48] was performed across all arrays, followed by linear model fitting using Limma [49]. Differentially expressed genes after 8 hours of RA treatment were defined by ranking all probesets by the moderated *t*-statistic-derived *P*-value (adjusted for multiple testing using Benjamini and Hochberg's method [50]) and setting thresholds of *P *< 0.01 and a fold-change of at least 2. All arrays were submitted to the NIH Gene Expression Omnibus (GEO) database under accession number [GEO:GSE19372].

### ChIP-seq protocols

ChIP protocols were adapted from [51]. Descriptions of these protocol modifications have been previously published [52]. Briefly, approximately 6 × 10e7 cells taken from each developmental time point were cross-linked using formaldehyde and snap-frozen in liquid nitrogen. Cells were thawed on ice, resuspended in 5 ml lysis buffer 1 (50 mM Hepes-KOH, pH 7.5, 140 mM NaCl, 1 mM EDTA, 10% glycerol, 0.5% NP-40, 0.25% Triton X-100) and mixed on a rotating platform at 4°C for 5 minutes. Samples were spun down for 3 minutes at 3,000 rpm, resuspended in 5 ml lysis buffer 2 (10 mM Tris-HCl, pH 8.0, 200 mM NaCl, 1 mM EDTA, 0.5 mM EGTA), and mixed on a rotating platform for 5 minutes at room temperature. Samples were spun down once more, resuspended in lysis buffer 3 (10 mM Tris-HCl, pH 8.0, 100 mM NaCl, 1 mM EDTA, 0.5 mM EGTA, 0.1% Na-deoxycholate, 0.5% N-lauroylsarcosine) and sonicated using a Misonix 3000 model sonicator to sheer cross-linked DNA to an average fragment size of approximately 500 bp. Triton X-100 was added to the lysate after sonication to final concentrations of 1% and the lysate spun down to pellet cell debris. The resulting whole-cell extract supernatant was incubated on a rotating mixer overnight at 4°C with 100 μl of Dynal Protein G magnetic beads that had been preincubated for 24 hours with 10 μg of the appropriate antibody in a phosphate-buffered saline/bovine serum albumin solution. Pan-RAR (Santa Cruz Biotechnology, Santa Cruz, CA, USA, sc-773), Pol2-S5P (Abcam, [Cambridge, UK, ab5131), and Pol2-S2P (Abcam, H5 clone ab24758) antibodies were used for ChIP experiments. After approximately 16 hours of bead-lysate incubation, beads were collected with a Dynal magnet. ChIP samples probing for TF binding were washed with the following regimen, mixing on a rotating mixer at 4°C for 5 minutes per buffer: low-salt buffer (20 mM Tris at pH 8.1, 150 mM NaCl, 2 mM EDTA, 1% Triton X-100, 0.1% SDS), high-salt buffer (20 mM Tris at pH 8.1, 500 mM NaCl, 2 mM EDTA, 1% Triton X-100, 0.1% SDS), LiCl buffer (10 mM Tris at pH 8.1, 250 mM LiCl, 1 mM EDTA, 1% deoxycholate, 1% NP-40), and TE containing 50 mM NaCl. ChIP samples probing for histone and chromatin marks were washed four times with RIPA buffer (50 mM Hepes-KOH, pH 7.6, 500 mM LiCl, 1 mM EDTA, 1% NP-40, 0.7% Na-deoxycholate) and then once with TE containing 50 mM NaCl, again mixing on a rotating mixer at 4°C for 5 minutes per buffer. After the final bead wash, samples were spun down to collect and discard excess wash solution, and bound antibody-protein-DNA fragment complexes were eluted from the beads by incubation in elution buffer at 65°C with occasional vortexing. Cross-links were reversed by overnight incubation at 65°C. Samples were digested with RNase A and Proteinase K to remove proteins and contaminating nucleic acids, and the DNA fragments precipitated with cold ethanol. Purified DNA fragments were processed according to a modified version of the Illumina/Solexa sequencing protocol [53].

Raw sequencing data (FASTQ format) were submitted to the NIH GEO/Sequence Read Archive database under accession number [GEO:GSE19409].

### Third-party ES cell ChIP-seq datasets

FASTQ files containing raw sequence and quality information were downloaded from the Short Read Archive [54].

The following published mouse ES cell experimental datasets were used to predict binding occupancy: c-Myc, CTCF, E2f1, Esrrb, Klf4, Nanog, n-Myc, Oct4, p300, Smad1, Sox2, STAT3, Suz12, Tcfcp2l1, Zfx, and green fluorescent protein control as published in Chen *et al. *[24]; Nanog, Oct4, Sox2, Tcf3, Suz12, H3K36me3, H3K4me3, H3K79me2, whole-cell extract (WCE) control as published in Marson *et al. *[37]; RNA-Pol2, H3K27me3, H3K36me3, H3K4me3, H3K9me3, H4K20me3, H3 control, WCE control as published in Mikkelsen *et al. *[38]; Ezh2, Ring1b, Suz12 as published in Ku *et al. *[39]; H3K4me1, H3K4me2 as published in Meissner *et al. *[40]; Brg, IgG control as published in Ho *et al. *[41].

Mouse ES cell Pol2-S5P and Pol2-S2P ChIP-seq experiments as published in Rahl *et al. *[36] were compared to Pol2 phosphorylation data generated by our study.

In addition, ChIP-seq experiments for other nuclear receptors were used in the construction of Additional file 1: mouse ES cell Nr5a2 as published in Heng *et al. *[26]; mouse adipocyte PPARγ and RXR as published in Nielsen *et al. *[25]; human MCF-7 ERα and RARα as published in Hua *et al. *[10].

### ChIP-seq data analysis

Sequence reads were aligned to the mouse genome (version mm8) using Bowtie [55] version 0.9.9.2 with options -k 2 --best. Only uniquely mapping reads were analyzed further. Multiple hits aligning to the same nucleotide position were discarded above the level expected at a 10^-7 ^probability from a per-base Poisson model of the uniquely mappable portion of the mouse genome. In practice, this caps the number of hits that start at the same nucleotide to three in the RAR ChIP-seq experiments.

Binding event detection for RAR, Pol2-S5P, and various published TF ChIP-seq experiments was carried out using a customized methodology that uses statistical significance testing to find regions producing an over-abundance of sequenced reads in the signal experiments compared with the control. The algorithm is run twice across the data. The first pass estimates a scaling factor for control sequencing read depth and a model of the distribution of sequencing read alignment hits around binding events. The second pass applies these parameters to predict a final set of significant events. Before the first pass, the scaling factor is initialized to be the ratio of total hit counts between the signal and control channels. The binding distribution model is initialized to be an empirical distribution estimated around predicted binding events in Oct4 ChIP-seq data [37] (Additional file 7).

All alignment hits are extended in both 3' and 5' directions, mirroring the observed distribution of hits around binding events. The extension magnitudes are set equal to the positions where the binding model distribution intersects a uniform distribution over the same area (Additional file 7). Control channel hit counts are scaled using the signal-control scaling factor. A sliding window of bin width 50 bp and offset 25 bp is run over the genome. Overlapping extended hit counts are calculated for both the signal and (scaled) control channels.

The background distribution of ChIP-seq hits is modeled as a non-homogenous Poisson process with parameters estimated from the scaled control hit counts. Specifically, the Poisson parameter λ is chosen as the maximum mean overlapping hit count in 50-bp windows of those observed from: i) the entire genome; ii) a 5-kbp window centered on the current location; and iii) a 10-kbp window centered on the current location. The use of this dynamic background model is motivated by the desire to correct local ChIP-seq enrichment biases that appear in the signal and control channels, and is similar to the model employed by MACS [56]. A given bin is denoted as potentially enriched if the overlapping hit count exceeds that expected from the background model at a *P*-value of 10^-9^.

*P*-values for each potentially enriched bin's over-representation in the signal channel over the control are calculated using the binomial distribution CDF [57]. Neighboring regions in the set of potentially enriched regions are merged, and the maximal *P*-value observed for the constituent bins is attached to the resulting merged region. The *P*-values are corrected for multiple hypothesis testing using Benjamini and Hochberg's method, and all regions with corrected *P*-values above 0.001 are discarded. False-discovery rates are estimated by repeating the event discovery procedures after swapping the scaled control channel and the signal channel.

After the first pass, the scaling factor is estimated by carrying out linear regression on the hit counts observed in 10,000-bp windows that are devoid of potentially significant events in both the signal and control channels. The binding model is estimated from enriched regions with *P*-values < 10^-7 ^and signal/control hit count enrichment > 10. These regions are aligned around the 'peak' location, defined as the position of maximum probability when scanning the current binding model over the region's hit landscape.

The above technique was also used when estimating enriched 'domains' for histone modifications, certain chromatin-associated proteins, and Pol2-S2P. However, to capture broader domains of enrichment, the bin width was set to 500 bp and the bin offset to 250 bp. The background model was also set to a homogeneous Poisson threshold estimated from the entire genome. The significance threshold was raised to *P *< 0.01 for domain calling.

### DNA motif analysis

*De novo *motif finding was performed in 200-bp windows centered on the 200 top-ranked peaks for each examined ChIP-seq experiment. SOMBRERO [58,59], AlignACE [60], BioProspector [61], and Weeder [62] were each run on the sequences. SOMBRERO was run for all even motif lengths between 8 and 22 bp with default settings apart from a complexity threshold of 0.01. A third-order Markov model of the mouse genome (version mm8) was employed as background, and a prior based on known mammalian TF binding motifs was also used [59]. AlignACE, BioProspector, and Weeder were run with default settings. STAMP [63] was used to cluster the discovered motifs and remove degeneracy in the results. STAMP was used to match two of the non-HRE motifs to the binding preference of Sp1, and to the 'M8' motif reported by Xie *et al. *[30] in a genome-wide scan of promoter sequences.

Log-likelihood scoring thresholds for the discovered DR5 and DR2 motifs were calculated by simulating 1,000,000 100-bp sequences using a third-order Markov model of the mouse genome (mm8 version). The motif scoring thresholds that yield false discovery rates of 1%, 0.5%, and 0.1% in this set of sequences were recorded.

The analysis of HRE motif frequency shown in Additional files 1 and 3 was performed using a model HRE half-site generated by aligning the half-sites in a curated database of confirmed HRE binding sites (NHRscan [64]). An arbitrary log-likelihood threshold of 5.0 was used to find matches to the half-site motif, which has the effect of matching the following half-site sequences that appear in the NHRscan database: TGACCT, TGACCC, TGAACT, TGTCCT, TGCCCT, TGAACC, TGGCCT, TGTCCC, TGATCT, TGACCA, TGACCG, TGCCCC, TGTACT, GGACCT, AGACCT, TGGCCC, TGAGCT, TCACCT, TGATCC, TAACCT, TGGACT. When scoring dimers, the same scoring threshold was used for both halves of the site, and the spacer sequence was unpenalized. These criteria are relatively strict; only 44% of confirmed HRE sites in the NHRscan database pass these thresholds.

### Comparison of binding site sets

A 200-bp window was used to define coincident locations between post-ES cell binding sites and ES cell binding sites or domains. The expected coincidence rates were calculated using a set of 10,000 randomly chosen genomic locations that are not located within 500 bp of any of the tested post-ES cell binding sites and also lie within 500-bp windows that are at least 80% uniquely mappable at a 26-mer resolution.

When calculating the rate of binding sites that are within 200 bp of at least one ES cell TF binding site, binding sites from the following 13 ES cell TF ChIP-seq experiments were used: the Young lab experiments for Oct4, Sox2, Nanog and Tcf3, and the Ng lab experiments for CTCF, c-Myc, n-Myc, E2f1, Esrrb, STAT3, Klf4, Smad1, and Zfx.

The clustergrams in Figures 1 and 5 were generated by plotting the overlapping read counts (where reads have been artificially extended to 200 bp) in 1-kbp windows centered on RAR peaks. The ordering of peaks was determined by clustering 50-bp-binned data using Matlab's clustergram function and the optimal leaf-ordering algorithm [65]. Since the various examined ChIP-seq experiments have different dynamic ranges and degrees of typical enrichment, a single color scale is not appropriate for all tracks. The saturation colors in Figure 5 are therefore chosen such that only 0.001% of the 50-bp windows in the genome will have overlapping read counts in excess (excluding those regions that have non-random accumulations of reads in sequenced control channels). The saturation thresholds for the tracks in Figure 5 are as follows: RAR (day 2 -RA) = 35, RAR (day 2 + RA) = 44 reads, H3K4me1 = 31 reads, H3K4me2 = 47 reads, H3K4me3 = 154 reads, Brg = 27 reads, c-Myc = 80 reads, CTCF = 71 reads, E2f1 = 489 reads, Esrrb = 329 reads, Klf4 = 57 reads, n-Myc = 67 reads, p300 = 18 reads, STAT3 = 50 reads, Tcfcp2l1 = 473 reads, Zfx = 92 reads, Nanog = 221 reads, Oct4 = 132 reads, Sox2 = 216 reads, Tcf3 = 125 reads.

### Constitutively bound and ligand-dependent RAR binding sites

A post-RA RAR binding site is defined as constitutively bound if it is within 200 bp of a significant binding site estimated in the pre-RA RAR experiment. As outlined in the main text, an RAR binding site is defined as being exclusively bound post-RA if it fulfills the criteria of being: i) significantly enriched post-RA in relation to the WCE control; ii) not significantly enriched pre-RA in relation to the WCE control; and iii) significantly enriched post-RA in relation to the pre-RA signal. The third criterion here entails performing peak-finding analysis for the post-RA RAR ChIP-seq experiment as described above, but substituting the pre-RA experiment for the WCE control. The aim is to ensure that a post-RA binding site does not display any ChIP-seq enrichment before RA exposure; for example, we wish to exclude events that display ChIP-seq enrichment just below the threshold of statistical significance in the pre-RA experiment. Equivalent procedures are carried out for finding condition-specific pre-RA RAR and Pol2-S5P binding sites.

### Motif specificity analysis

The motif specificity analysis presented in Figure 6 is based on genome-wide matches to the DR2 or DR5 motifs. A scoring threshold for these motifs was chosen such that one-third of all RAR (day 2 +RA) peaks had a match to either motif within 100 bp of the peak position. These criteria yield 929 'true-positive' hits to the DR2/5 motifs proximal to 644 peaks. The same scoring thresholds yield over 877,000 matches to the DR2/5 motifs throughout the mouse genome. This set of positions was filtered for those that do not overlap RAR binding events (within 200 bp) and are located within 500-bp windows that are at least 80% uniquely mappable at a 26 bp resolution. Thus, the set of 'additional predictions' contains 582,612 positions. Some of these additional predictions may serve as binding sites for other TFs, or indeed for RAR under different cellular conditions. However, the vast majority are expected to be false positive predictions.

The log-likelihood similarity score to DR2/5 motifs was used to rank the predictions, and the additional prediction rate for each true positive rate is plotted in Figure 6. Next, for various ES ChIP-seq experiments, the number of reads contained in a 500-bp window surrounding each motif match was counted, and these counts were normalized according to the total uniquely mapped read count for the experiment of interest. For each motif match, normalized read counts were summed across two collections of experiments; the ES cell TF collection (the 16 sequence-specific TFs) and the ES cell H3K4 collection (H3K4me1/2/3 from the Bernstein lab, and H3K4me3 from the Young lab). The motif matches were then ranked separately for each collection according to the summed normalized read counts. Average phastCons [66] conservation scores were also calculated for a 20-bp window around each motif match, and this scoring was again used for ranking (50-bp, 100-bp, and 200-bp windows were also tested for generating phastCons scores, without any increase in performance). To generate a combined ranking for two data sources (for example, motif + ES cell TFs), we normalized the ranking such that each motif match was assigned a score between 0 and 1 for each data source. The product of both scores is taken for each motif match, and the matches are again ranked based on this score. Again, the additional prediction rate for each true positive rate is plotted in Figure 6 for each combined score. We note that the described method for combining motif and ES cell data scores is simplistic, and more sophisticated schemas for incorporating knowledge of ES cell ChIP-seq data may attain much greater improvements in motif specificity than presented in Figure 6.

### Support vector machine classification of sites

SVMs were implemented using the libSVM R library [67], and trained using default settings (C-classification, RBF kernels). Positive training sets were generated by randomly selecting 500 bound sites from each set of predicted peaks. A negative set of 10,000 randomly chosen sites was also defined. The negative set sites are located within 500-bp windows that are at least 80% uniquely mappable at a 26-bp resolution, and do not overlap any sites bound by the test TFs (that is, RAR, PPARγ, Foxa2). ChIP-seq read counts were extracted from 500-bp windows surrounding the positive and negative positions. During each SVM training run, 50 positive sites and 50 negative sites were randomly extracted as test data. The SVMs were trained on the remaining data, and predictive performance was tested on the held out data. SVM training was repeated 100 times, and the averages of the resulting receiver operating characteristic (ROC)-area under curves (AUC) values are reported in Table 1.

## Abbreviations

bp: base pair; ChIP: chromatin immunoprecipitation; DR: direct-repeat motif; ER: estrogen receptor; ES: embryonic stem cell; GEO: Gene Expression Omnibus; HRE: hormone response element; kbp: kilo-base-pair; Pol2: RNA polymerase II; Pol2-S2P: Pol2 CTD serine 2 phosphorylation; Pol2-S5P: Pol2 CTD serine 5 phosphorylation; PPAR: peroxisome proliferator activated receptor; RA: retinoic acid; RAR: retinoic acid receptor; RARE: retinoic acid response element; SVM: support vector machine; TF: transcription factor; WCE: whole-cell extract.

## Authors' contributions

All authors participated in the design of the study. EOM performed cell culture, motor neuron differentiation, and gene expression profiling. SMcC performed the ChIP-seq experiments. SM designed and performed all computational analyses. SM, EOM, HW and DKG drafted the manuscript. All authors read and approved the final manuscript.

## Supplementary Material

Additional file 1 **Supplementary Figure S1**. Enrichment of HRE motifs with various configurations and half-site spacer lengths under ChIP-enriched regions for various nuclear hormone receptor TFs [10,24-26]. The bar-charts show the frequencies of peaks containing each HRE motif configuration within 50 bp of the top 20% of binding sites using strict similarity to a model HRE half-site (see Materials and methods). Click here for file

Additional file 2 **Supplementary Figure S2**. Higher-ranked RAR binding sites are more likely to contain DR2/5 motifs. The figure shows the cumulative proportion of ranked peaks that contain matches to the DR2 or DR5 motifs at a threshold set using the 0.5% false positive rate. Click here for file

Additional file 3 **Supplementary Figure S3**. Enrichment of HRE motifs with various configurations and half-site spacer lengths at the top 100 constitutive and ligand-specific RAR binding sites (as in Additional file 1). Click here for file

Additional file 4 **Supplementary Figure S4**. RAR binding shifts in response to retinoic acid exposure. The plots in the two leftmost columns show enrichment over all constitutive and ligand-specific RAR binding sites (± 1 kbp over the binding site), where the blue shading corresponds to the ChIP-seq read count in the region. The plots to the right show matches to motifs over the same regions, with three motif similarity thresholds represented by green color shading (0.5% false positive rate motif scoring thresholds). Click here for file

Additional file 5 **Supplementary Table S1**. Differential frequencies of motifs in exclusively post-RA binding sites compared with exclusively pre-RA binding sites, and vice versa. Only motifs with a *P*-value < 0.05 are shown. The motif names have prefixes denoting their source, as follows: T = TRANSFAC, J = Jaspar, X = Xie *et al. *[30], U = UniProbe. Click here for file

Additional file 6 **Supplementary Table S2**. List of 96 differentially expressed genes (> 2-fold, *P *< 0.01) between day 2 + 8 hours RA and day 2. Tick marks denote the presence of RAR binding sites within 20 kbp of a gene's transcription start site in the presence or absence of RA. Click here for file

Additional file 7 **Supplementary Figure S5**. Empirically estimated distribution of Oct4 ChIP-seq hits around predicted peaks. The uniform expectation over the same area is shown as a dashed red line. Click here for file
