# Supplementary material for: Ligand-dependent dynamics of retinoic acid receptor binding during early neurogenesis
Source: Genome Biol. 2011 Jan 13;12(1):R2. doi: 10.1186/gb-2011-12-1-r2 (PMC3091300; doi:10.1186/gb-2011-12-1-r2)
Supplement: Additional file 5 — Supplementary Table S1. Differential frequencies of motifs in exclusively post-RA binding sites compared with exclusively pre-RA binding sites, and vice versa. Only motifs with a P-value < 0.05 are shown. The motif names have prefixes denoting their source, as follows: T = TRANSFAC, J = Jaspar, X = Xie et al. [30], U = UniProbe. [file gb-2011-12-1-r2-S5.doc]

**Supplementary Table S**1

| **RAR Day2 +RA exclusive motifs** | | | | | | |  |  |
| --- | --- | --- | --- | --- | --- | --- | --- | --- |
| *Motif name* | *Percentage of RAR Day2+RA exclusive peaks containing motif (threshold=1% FP)* | | *Percentage of RAR Day2-RA exclusive peaks containing motif (threshold=1% FP)* | | | *Over-representation* | *P-value* | *Motif* |
| J_MA0329.1_MBP1 | 9.4% | | 2.2% | | | 4.22 | 1.42E-04 | 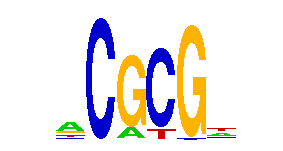 |
| M8-like | 7.8% | | 2.4% | | | 3.25 | 8.81E-03 | 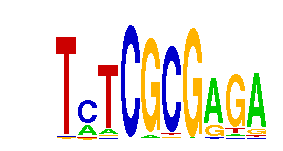 |
| J_MA0098.1_ETS1 | 8.8% | | 3.0% | | | 2.96 | 1.06E-02 | 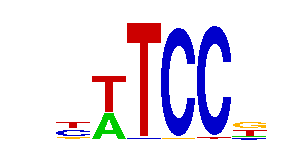 |
| T_STAT1_M00492 | 5.2% | | 1.1% | | | 4.65 | 1.09E-02 | 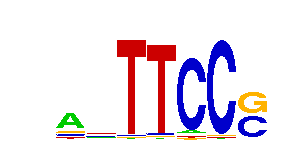 |
| X_M4 | 6.3% | | 1.7% | | | 3.75 | 1.10E-02 | 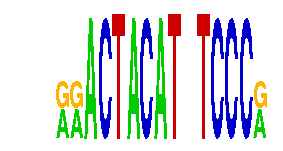 |
| DR5 | 13.2% | | 6.3% | | | 2.09 | 1.10E-02 | 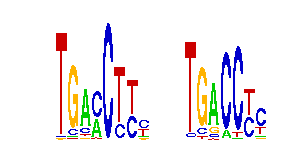 |
| T_StuAp_M00263 | 10.5% | | 4.3% | | | 2.46 | 1.13E-02 | 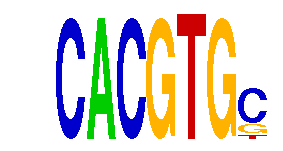 |
| T_bHLH66_M01054 | 5.6% | | 1.3% | | | 4.34 | 1.17E-02 | 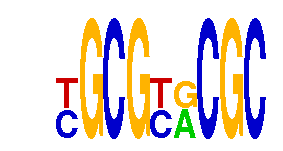 |
| X_M30 | 14.3% | | 7.2% | | | 1.97 | 1.18E-02 | 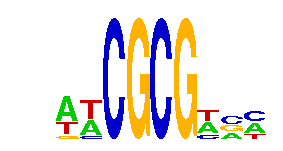 |
| T_Elk-1_M00025 | 8.2% | | 3.0% | | | 2.75 | 1.24E-02 | 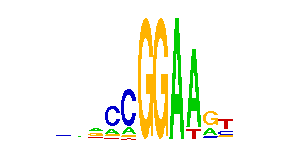 |
| T_E2F_M00803 | 18.2% | | 10.4% | | | 1.75 | 1.26E-02 | 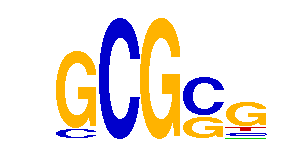 |
| T_E2F_M00920 | 6.9% | | 2.2% | | | 3.10 | 1.26E-02 | 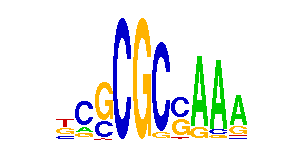 |
| U_Yll054c-primary | 10.2% | | 4.5% | | | 2.29 | 1.29E-02 | 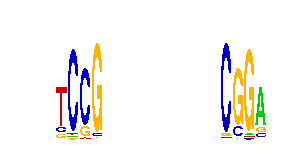 |
| X_M2 | 6.4% | | 2.0% | | | 3.15 | 1.45E-02 | 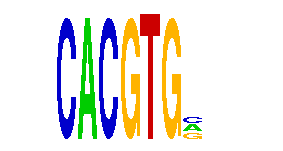 |
| T_CLOCK-BMAL_M01116 | 5.2% | | 1.3% | | | 3.98 | 1.50E-02 | 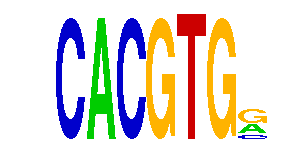 |
| J_MA0353.1_PDR3 | 14.7% | | 8.0% | | | 1.85 | 1.64E-02 | 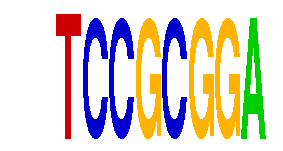 |
| T_HBP-1b_M00697 | 4.4% | | 0.9% | | | 4.73 | 1.66E-02 | 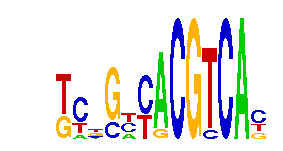 |
| J_MA0361.1_RDS1 | 10.2% | | 4.6% | | | 2.20 | 1.66E-02 | 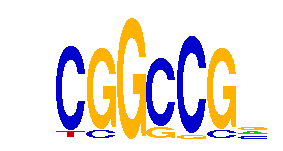 |
| J_MA0016.1_usp | 8.8% | | 3.7% | | | 2.37 | 1.88E-02 | 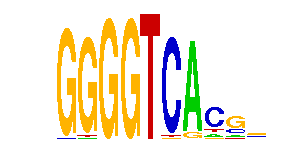 |
| T_Staf_M00264 | 5.5% | | 1.7% | | | 3.29 | 2.36E-02 | 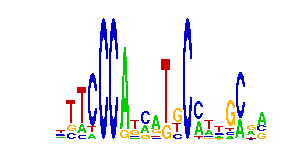 |
| T_E2F_M00516 | 5.8% | | 1.9% | | | 3.13 | 2.43E-02 | 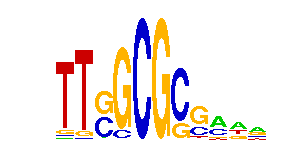 |
| U_Rsc3-primary | 10.0% | | 4.8% | | | 2.08 | 2.89E-02 | 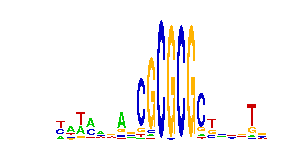 |
| U_Rsc30-primary | 16.5% | | 9.8% | | | 1.67 | 2.97E-02 | 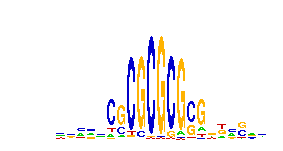 |
| J_MA0374.1_RSC3 | 10.8% | | 5.4% | | | 2.01 | 2.99E-02 | 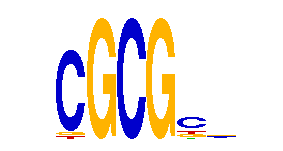 |
| T_CF1_M00112 | 7.8% | | 3.3% | | | 2.35 | 3.16E-02 | 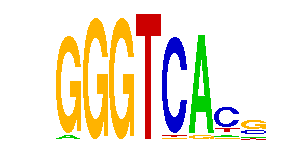 |
| J_MA0311.1_HAL9 | 12.5% | | 6.9% | | | 1.83 | 3.56E-02 | 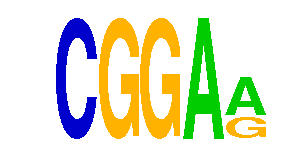 |
| J_MA0159.1_RXR_RAR_DR5 | 12.1% | | 6.5% | | | 1.86 | 3.60E-02 | 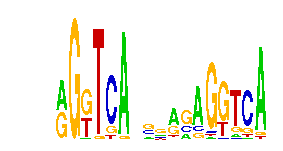 |
| U_Hal9-primary | 9.7% | | 4.8% | | | 2.01 | 4.02E-02 | 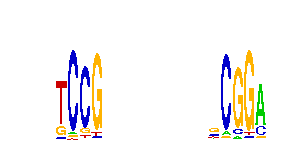 |
| J_MA0375.1_RSC30 | 11.9% | | 6.5% | | | 1.83 | 4.06E-02 | 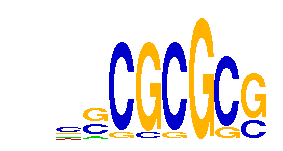 |
| T_c-Ets-1_M01078 | 5.6% | | 2.0% | | | 2.76 | 4.18E-02 | 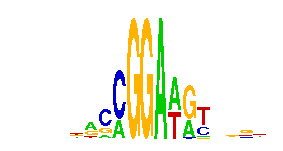 |
| J_MA0280.1_CAT8 | 9.7% | | 4.8% | | | 2.01 | 4.27E-02 | 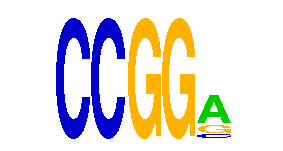 |
| J_MA0076.1_ELK4 | 5.6% | | 2.0% | | | 2.76 | 4.30E-02 | 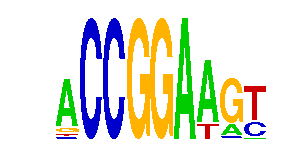 |
|  |  | |  | | |  |  |  |
| **RAR Day2 exclusive motifs** | | | | | |  |  |  |
| *Motif name* | | *Percentage of RAR Day2+RA exclusive peaks containing motif (threshold=1% FP)* | | *Percentage of RAR Day2 exclusive peaks containing motif (threshold=1% FP)* | | *Over-representation* | *P-value* | *Motif* |
| T_LRH1_M01142 | 6.9% | | | | 1.9% | 3.65 | 4.65E-03 | *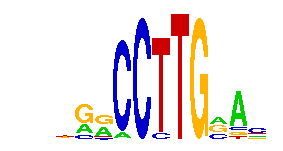* |
| J_MA0379.1_SIG1 | 9.3% | | | | 3.3% | 2.82 | 5.37E-03 | 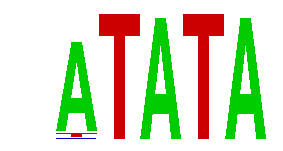 |
| T_GCNF_M00526 | 7.6% | | | | 2.2% | 3.47 | 5.43E-03 | 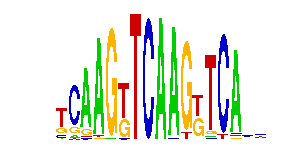 |
| DR0 | 8.9% | | | | 3.4% | 2.58 | 1.49E-02 | 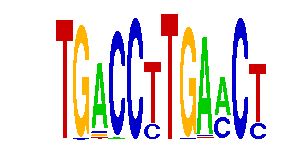 |
| J_MA0094.1_Ubx | 35.6% | | | | 25.2% | 1.41 | 1.66E-02 | 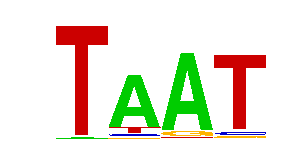 |
| J_MA0141.1_Esrrb | 9.6% | | | | 4.1% | 2.37 | 1.71E-02 | 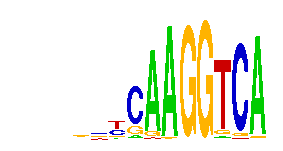 |
| T_SF1_M01132 | 6.5% | | | | 2.4% | 2.76 | 4.15E-02 | 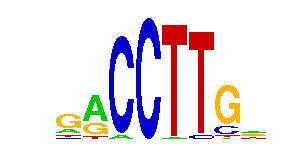 |
| J_MA0218.1_ct | 10.0% | | | | 4.7% | 2.13 | 4.29E-02 | 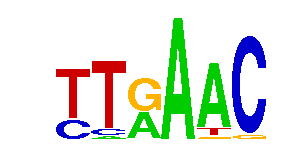 |
| J_MA0071.1_RORA_1 | 6.3% | | | | 2.2% | 2.87 | 4.39E-02 | 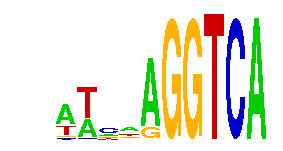 |
| J_MA0115.1_NR1H2_RXRA | 8.5% | | | | 3.8% | 2.27 | 4.75E-02 | 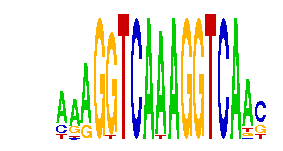 |
