# Supplementary material for: Ligand-dependent dynamics of retinoic acid receptor binding during early neurogenesis
Source: Genome Biol. 2011 Jan 13;12(1):R2. doi: 10.1186/gb-2011-12-1-r2 (PMC3091300; doi:10.1186/gb-2011-12-1-r2)
Supplement: Additional file 6 — Supplementary Table S2. List of 96 differentially expressed genes (> 2-fold, P < 0.01) between day 2 + 8 hours RA and day 2. Tick marks denote the presence of RAR binding sites within 20 kbp of a gene's transcription start site in the presence or absence of RA. [file gb-2011-12-1-r2-S6.doc]

**Supplementary Table S2**

| **Probe** | **Foldchange** | **P-value** | **Day2-RA RAR binding site within 20Kbp of TSS** | **Day2+RA RAR binding site within 20Kbp of TSS** |
| --- | --- | --- | --- | --- |
| Zfp703 | 7.985 | 8.41E-07 |  |  |
| Hoxb5 | 7.738 | 8.49E-07 |  | ✓ |
| Hoxb1 | 7.569 | 6.55E-06 |  | ✓ |
| Hoxb4 | 5.492 | 3.61E-05 |  | ✓ |
| Cyp26a1 | 6.667 | 0.000111171 | ✓ | ✓ |
| Hoxb2 | 4.070 | 6.90E-05 |  | ✓ |
| Hoxa1 | 5.557 | 1.98E-06 | ✓ | ✓ |
| Hoxa4 | 4.530 | 5.92E-05 |  | ✓ |
| Hoxa5 | 4.983 | 5.79E-07 |  | ✓ |
| Cdx1 | 5.436 | 6.65E-28 | ✓ | ✓ |
| Hoxa3 | 5.173 | 0.001039013 |  | ✓ |
| Stra8 | 5.400 | 1.07E-05 | ✓ | ✓ |
| Hoxb6 | 4.701 | 0.001731881 |  | ✓ |
| Hoxb3 | 3.858 | 0.000125622 |  | ✓ |
| Meis2 | 4.106 | 4.07E-05 | ✓ | ✓ |
| Glra2 | 4.335 | 6.47E-07 |  |  |
| Dhrs3 | 4.163 | 0.004373169 |  |  |
| Hoxc4 | 3.873 | 6.47E-07 |  |  |
| Tshz1 | 3.535 | 0.004358244 |  | ✓ |
| Zadh2 | 3.827 | 0.002345752 |  | ✓ |
| Cnnm2 | 3.642 | 0.000649467 |  |  |
| Cpvl | 3.560 | 5.09E-06 |  |  |
| Hoxa2 | 3.536 | 0.000321083 | ✓ | ✓ |
| Kcnh1 | 3.533 | 0.000212552 |  |  |
| Rarb | 3.523 | 0.000112082 | ✓ | ✓ |
| E130309F12Rik | 3.275 | 0.000228566 |  |  |
| 6330442E10Rik | 3.285 | 0.009263349 |  | ✓ |
| Nrip1 | 2.977 | 7.21E-05 |  |  |
| Zfp503 | 2.967 | 0.009475262 |  |  |
| 5730446D14Rik | 2.963 | 0.000127303 | ✓ | ✓ |
| Hoxa10 | 2.908 | 0.000112082 |  |  |
| Fbp1 | 2.857 | 0.001427658 |  |  |
| Kit | 2.178 | 0.003686133 |  |  |
| Ankrd43 | 2.716 | 5.31E-05 | ✓ | ✓ |
| Nt5e | 1.985 | 0.004916707 |  |  |
| Ednrb | 2.651 | 2.51E-06 |  |  |
| Wdr40b | 2.615 | 1.52E-06 |  |  |
| Nr0b1 | 2.566 | 0.000563019 |  |  |
| Rec8 | 2.558 | 0.002178472 | ✓ | ✓ |
| Folr4 | 2.408 | 0.000128905 |  |  |
| AK220484 | 2.314 | 0.004844237 | ✓ | ✓ |
| Fzd4 | 2.274 | 0.000321083 |  |  |
| Zmym3 | 2.271 | 0.000188994 |  |  |
| Amhr2 | 2.257 | 2.17E-05 |  |  |
| Ccdc88b | 2.244 | 4.06E-08 | ✓ | ✓ |
| Ascl1 | 2.210 | 0.002564943 |  |  |
| Tspyl4 | 2.200 | 0.00727743 | ✓ | ✓ |
| Agpat3 | 1.628 | 0.001275563 | ✓ | ✓ |
| Rbp1 | 2.101 | 0.00433861 | ✓ |  |
| A330049M08Rik | 2.076 | 0.000469823 | ✓ | ✓ |
| Rasl10b | 1.992 | 0.00433861 | ✓ | ✓ |
| C1galt1 | 1.950 | 0.000533147 |  |  |
| Plekha6 | 1.814 | 0.000167402 |  |  |
| Fndc5 | 1.790 | 0.000711323 | ✓ | ✓ |
| 3110037C07Rik | 1.428 | 1.27E-05 | ✓ |  |
| Cphx | 1.692 | 0.001731881 |  |  |
| Hcfc2 | 1.686 | 0.006171106 |  |  |
| Tmem132e | 1.678 | 0.008385155 |  |  |
| Letmd1 | 1.389 | 0.001017317 | ✓ | ✓ |
| Art1 | 1.439 | 0.001175643 |  |  |
| Cxcl12 | 1.424 | 0.003566147 |  |  |
| Cd14 | 1.422 | 0.00066503 |  | ✓ |
| Apob48r | 1.415 | 0.000179167 |  | ✓ |
| Tmtc1 | 1.364 | 0.001232436 |  |  |
| Wnt8a | 1.332 | 8.11E-07 |  |  |
| Tmem38b | 1.328 | 0.00013972 |  |  |
| Meis1 | 1.293 | 0.001332218 |  |  |
| Gtsf1 | 1.205 | 0.002216022 |  |  |
| Btn1a1 | 1.201 | 0.003369589 | ✓ | ✓ |
| Nlrp4f | 1.179 | 0.000649467 |  |  |
| Nat1 | 1.169 | 1.58E-05 |  |  |
| Efnb1 | 1.138 | 0.007347157 |  |  |
| Akr1c19 | 1.135 | 0.001602599 |  |  |
| Upb1 | 1.127 | 0.000815518 |  |  |
| Cad | 1.111 | 1.98E-06 |  |  |
| Tbxa2r | 1.106 | 6.47E-07 |  |  |
| Fndc1 | 1.073 | 0.002199996 |  |  |
| Pbx2 | 1.045 | 0.001276976 | ✓ | ✓ |
| Tradd | 1.003 | 0.001135875 |  | ✓ |
| Aoah | -1.064 | 0.00398022 |  |  |
| Enc1 | -1.080 | 0.001232436 |  |  |
| Tmem100 | -1.230 | 0.002609969 |  |  |
| Fgf4 | -1.468 | 2.89E-05 |  |  |
| Cbr3 | -1.377 | 0.003566147 |  |  |
| Ttyh1 | -1.404 | 0.003194157 |  |  |
| Mycn | -1.670 | 0.002329768 |  |  |
| Tnfsf11 | -1.738 | 0.008949815 |  |  |
| Cdc42ep5 | -1.781 | 0.001864911 |  |  |
| Calcr | -1.866 | 0.000623112 |  |  |
| Mlstd1 | -2.097 | 0.000188994 |  |  |
| Aadat | -2.188 | 0.000507853 |  |  |
| Eomes | -3.113 | 0.002113039 |  |  |
| Fst | -2.747 | 0.001944027 |  |  |
| Glod5 | -2.940 | 0.001438989 |  |  |
| Fgf5 | -3.473 | 5.09E-06 |  |  |
| Otx2 | -4.389 | 8.61E-05 |  |  |
